# Promoting public skin health through a national continuing medical education project on cosmetic and dermatologic sciences: a 15-year experience

**DOI:** 10.3389/fpubh.2023.1273950

**Published:** 2023-11-16

**Authors:** Yiming Li, Xiaohong Shu, Wei Huo, Xi Wang

**Affiliations:** ^1^Department of Dermatology and Venerology, West China Hospital, Sichuan University, Chengdu, China; ^2^Laboratory of Dermatology, Clinical Institute of Inflammation and Immunology (CIII), Frontiers Science Center for Disease-related Molecular Network, West China Hospital, Sichuan University, Chengdu, China; ^3^Center of Cosmetics Evaluation, West China Hospital, Sichuan University, Chengdu, China

**Keywords:** public skin health, cosmetic sciences, dermatologic sciences, continuing medical education, online survey

## Abstract

**Background:**

The developments in cosmetic sciences and technologies have generated a gap between the cosmetics and their users. Users including regular customers, clinicians, industry personnel, researchers, testing agencies, beauty salon workers, and mass media hardly possess the ability to distinguish truth from falsehood. The gap remained as one major reason for inappropriate cosmetics usage, insufficient efficacy, and even cosmetics adverse reactions (CARs).

**Methods:**

Aiming at enhancing the relevant practitioners’ cosmetic and dermatologic sciences, we launched a cosmetic and dermatologic sciences continuing medical education (CME) since 2008. The objective of the current study was to evaluate the effectiveness of the CME. We summarized and analyzed the project for the last 15 years. Meanwhile, an online survey consisted of three parts was performed to evaluate the CME and to collect the trainees’ comments.

**Results:**

A total of 3,923 trainees have participated in the CME project from 2008 to 2022. The trainees included clinicians, industry staffs, biomedical researchers, third-party cosmetics testing staffs, beauty salon staffs, students, and media staffs. The trainees had theory courses on cosmetic and dermatologic sciences, cosmetics DIY practice & video watching, and an optional guided tour during the 4.5-day CME. Eight hundred and twenty-three trainees and 586 control subjects responded to the online survey. The comprehensive test in the second part of the survey demonstrated that compared with the control group, the CME project significantly enhanced the trainees’ perception and knowledge regarding the cosmetics formula sciences, basic dermatologic sciences, cosmetics usage, noninvasive measurements, new advances, CARs, and laws (*p* = 0.000). Trainees of all occupations ranked “basic dermatologic sciences and skin diseases” as the most significant sections. Trainees of all occupations believed the CME has contributed most in “understand the function & efficacy of cosmetics.” We noticed the occupational variances. Over 97% of trainees were willing to recommend the CME to the others.

**Conclusion:**

The CME project significantly enhanced the trainees’ cosmetic and dermatologic sciences, which bridged the gap between cosmetics and public skin health. This multidisciplinary CME also contributed to establishing an interdisciplinary interaction and cooperation platform for the multiple occupations involved in the public skin health maintenance and promotion.

## Introduction

The unprecedented developments in sciences and technologies have contributed to a fast-growing and expanding cosmetics market in China. Been increasingly involved in our daily life, cosmetics have been utilized to clean, improve or change the appearance of skin, hair, nails, and teeth (1). The usage of cosmetics also proved to be beneficial for a variety of pathological conditions. Significant symptoms alleviation and long-lasting clinical relief could be achieved with the adjuvant therapy of proper cosmetics for inflammatory skin diseases, such as atopic dermatitis, contact dermatitis, psoriasis, rosacea, acne, and seborrheic dermatitis (2–11). The application of functional cosmetics is believed to be positively effective in dyspigmentation and disfiguring dermal diseases management as well (12–16).

Nevertheless, the ascending incidence of cosmetics adverse reactions (CARs) has been noticed and documented. Inappropriate selection, application, and removing of cosmetics might damage the skin barrier and induce dermatopathological symptoms (17–19). Cosmetics-related facial eczema, allergic contact dermatitis, contact urticaria, photosensitivity, acne, discoloration, and hair and nail diseases might be associated with the functional ingredients, fragrances, preservatives in cosmetics ranging from cleansers to makeups (20–23). Products containing illegal components, for instance, heavy metals and mercury, can be potentially risky to the users (24–26). The CARs may occur in healthy population and even cases under immunosuppressive therapy (27), which jeopardize the public dermatologic health.

Cosmetics enterprises, efficacy and safety evaluation agencies, dermatologists, beauty salons, and academic researchers are indispensable parts involved in the cosmetics-public dermatologic health interaction. Enhancing their cosmetic and dermatologic sciences may contribute substantially to products research and development (R&D), cosmetics selection and application, CARs prevention, identification, and management. Although cosmetic sciences have been lectured in certain universities (28), the majority of trainees are students, especially undergraduate ones. The aforementioned indispensable parts involved in the cosmetics-public skin health interaction, however, were not covered and educated.

Aiming at enhancing the relevant practitioners’ cosmetic and dermatologic sciences, we organized and launched a cosmetic and dermatologic sciences education program since 2008. This program has been listed as a national continuing medical education (CME) project by the Chinese Medical Association since 2008. The objective of the current study was to evaluate the effectiveness of the education program. We summarized and analyzed the project for the last 15 years. Meanwhile, an online survey was performed to evaluate the CME project and to collect the trainees’ comments.

## Methods

### The CME project on cosmetic and dermatologic sciences

This 4.5-day structured education program was comprised of theory courses, do it yourself (DIY) practice & video watching, and an optional visiting tour. The curriculum was the same for all participants. Courses contents were designed based upon the broad range of participants’ backgrounds and covered a wide range of areas. The participants may choose the courses according to their preference, and an attendance rate of 80% were required for taking the exam at the end of the CME. The exam consisted of multiple choices and true or false questions. Those who scored over 60/100 received the national CME credits. We established a Tecent QQ group or WeChat group for the trainees each year for liaison and issuing news and advances in the relevant field.

### Online survey

A preliminary survey (Supplementary material 1) was sent to the CME participants to investigate whether they were willing to attend the online survey, and to collect the relevant professional background information. An online survey was then utilized via the Wenjuanxing website^1^ from January 2023 to March 2023. The trainees’ colleagues or classmates with similar education level and work experience, but without attending this CME project or similar ones, were invited as a control group.

The online survey was comprised of 3 parts (Supplementary material 2). The first part included the basic information survey. The second part was a comprehensive test consisted of 25 single-choice questions on cosmetic and dermatologic sciences, which was required to completed within 25 min. The third part was to collect the trainees’ comments on the CME project. Both the trainee group and the control group were required to complete the first and the second parts. Only the trainee group needed to complete the third part. The ethical committee of West China hospital, Sichuan University approved this survey (no. 2023-404). Informed consent was received from each of the participants.

### Statistical analysis

All quantitative data were expressed as mean ± SD. All categorical data were presented using frequencies or proportions. Analysis was conducted with SPSS 23 (SPSS, Inc., Chicago, IL). The comparison of ratios was performed by the use of Mann–Whitney U test or Fisher exact test. A value of *p* < 0.05 was considered statistically significant.

## Results

### The CME project and trainees

**Figure 1:**
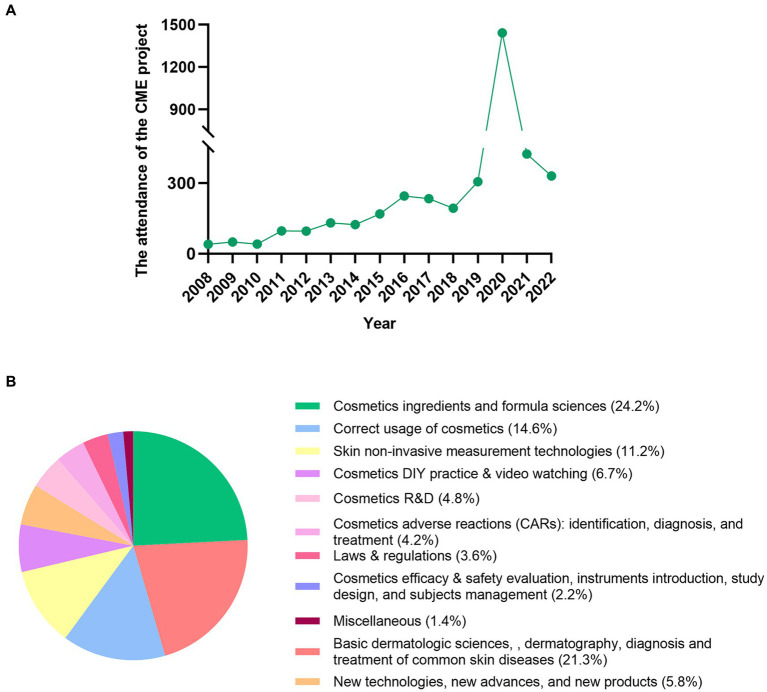
The attendance of the CME project and courses sections. **(A)** the attendance of the CME project and **(B)** the 11 courses sections.

A total of 3,923 trainees have participated in the CME project from 2008 to 2022. The maximal attendance was 1,443 in 2020, when the CME project was online due to the COVID-19 pandemic (Figure 1A). The CME project was organized both offline and online starting 2021. The trainees included clinicians, industry staffs, biomedical researchers, third-party cosmetics efficacy & safety testing staffs, beauty salon staffs, undergraduate or graduate students, and media staffs.

The trainees attended theory courses on the first 3 days. The theory courses focused on cosmetic and dermatologic sciences. The trainees had the cosmetics DIY practice & video watching on the 4th day. They learned to prepare cosmetics such as moisturizers, cleansers, lipsticks, and shampoos with miscellaneous ingredients or compounds. The videos mainly covered regular aesthetic medicine procedures such as chemical peeling, energy devices, and botulinum toxin & filler injection. The theory courses and DIY practice & video watching lasted for 40–45 min per class. Renowned professionals with the relevant specialties lectured the courses (Figure 1B; Table 1). The teaching materials were the printed PPTs and supplementary scientific brochures.

**Table 1:** The CME courses sections and lecturers.

Sections	Lecturer (s)
Cosmetics ingredients and formula sciences	Cosmetics industry R&D staff, light industrial designing institute engineers, pharmacists, dermatologists
Basic dermatologic sciences, dermatologic imageology, diagnosis and treatment of common skin diseases	Dermatologists, biomedical researchers
Correct usage of cosmetics	Dermatologists
Skin non-invasive measurement technologies	Third-party cosmetics efficacy & safety testing staff, cosmetics industry Efficacy & safety evaluation staff, dermatologists
Cosmetics DIY practice & video watching	Cosmetics industry R&D staff, dermatologists
New technologies, new advances, and new products	Cosmetics industry R&D staff, dermatologists, biomedical researchers
Cosmetics R&D	Cosmetics industry R&D staff, biomedical researchers, dermatologists
CARs: identification, diagnosis, and treatment	Dermatologists
Laws & regulations	State food and drug administration (SFDA) staff
Cosmetics efficacy & safety evaluation: instruments introduction, study design, and subjects management	Cosmetics efficacy & safety testing technicians, instruments manufacturers, dermatologic bioengineering researchers
Miscellaneous	Depends on the specific content

On the 5th day, the trainees had the optional guided tour to the outpatient section, dermatologic surgery section, dermatologic pathology section, medical aesthetics section, and ward at the department of dermatology and venerology, West China hospital.

### Outcomes of the online survey

For the trainee group, we sent out 3,923 preliminary surveys and received 854 responses indicating the willingness to attend the online survey (response rate 21.8%). We then sent out 854 online surveys and received 823 valid responses (response rate 96.4%). For the control group, we sent out 904 online surveys and received 586 valid responses (response rate 64.8%).

The professional characteristics were listed in Tables 2–4. The age and gender distribution of the participants in both groups was listed in Supplementary material 3. For both groups, clinicians were from departments of dermatology & venerology, cosmetic aesthetics, and traditional medicine. The industry staffs included R&D staffs, efficacy & safety evaluation staffs, sales staffs, and management staffs.

**Table 2:** Professional characteristics of the on-line survey respondents (clinicians, cosmetics industry staffs, and third-party cosmetics efficacy & safety testing staffs).

		Clinicians			Cosmetics industry staffs			Third-party cosmetics efficacy & safety testing staffs		
		Trainee group *N* = 373	Control group *N* = 195	*Z* or Fisher; *p* values	Trainee group *N* = 260	Control group *N* = 191	*Z* or Fisher; *p* values	Trainee group *N* = 80	Control group *N* = 72	*Z* or Fisher; *p* values
Education level, *n* (%)	Doctor’s degree	39 (10.5)	14 (7.2)	−0.87; 0.385	9 (3.5)	2 (1)	−0.891; 0.373	3 (3.8)	–	4.488; 0.335
	Master’s degree	147 (39.4)	84 (43)		80 (30.8)	66 (34.6)		29 (36.3)	26 (36.1)	
	Bachelor’s degree	166 (44.5)	75 (38.5)		145 (55.8)	90 (47.1)		45 (56.3)	40 (55.6)	
	College diploma	18 (4.8)	20 (10.3)		24 (9.2)	30 (15.7)		3 (3.8)	4 (5.6)	
	Below college	3 (0.8)	2 (1)		2 (0.7)	3 (1.6)		–	2 (2.8)	
Work experience (yrs), *n* (%)	≤5	69 (18.5)	22 (11.3)	−1.668; 0.095	103 (39.6)	95 (49.7)	−0.79; 0.43	48 (60)	54 (75)	−1.67; 0.095
	6–9	78 (20.9)	54 (27.7)		72 (27.7)	33 (17.3)		16 (20)	7 (9.7)	
	10–19	135 (36.2)	55 (28.2)		74 (28.5)	40 (20.9)		13 (16.3)	6 (8.3)	
	≥20	91 (24.4)	64 (32.8)		11 (4.2)	23 (12)		3 (3.8)	5 (6.9)	

**Table 3:** Professional characteristics of the on-line survey respondents (Biomedical researchers, beauty salon staffs, and media staffs).

		Biomedical researchers			Beauty salon staffs			Media staffs		
		Trainee group *N* = 42	Control group *N* = 38	*Z* or Fisher; *p* values	Trainee group *N* = 37	Control group *N* = 41	*Z* or Fisher; *p* values	Trainee group N = 9	Control group *N* = 14	*Z* or Fisher; *p* values
Education level, *n* (%)	Doctor’s degree	16 (38.1)	10 (26.3)	2.307; 0.506	–	–	3.27; 0.314	–	–	0.639; 0.824
	Master’s degree	16 (38.1)	18 (47.4)		1 (2.7)	–		1 (11.1)	3 (21.4)	
	Bachelor’s degree	10 (23.8)	9 (23.7)		15 (40.5)	11 (26.8)		7 (77.8)	9 (64.3)	
	College diploma	–	1 (2.6)		13 (35.1)	16 (39)		1 (11.1)	2 (14.3)	
	Below college	–	–		8 (21.6)	14 (34.1)		–	–	
Work experience (yrs), *n* (%)	≤5	13 (31)	15 (39.5)	−1.155; 0.248	12 (32.4)	17 (41.5)	3.496; 0.322	3 (33.3)	5 (35.7)	1.842; 0.743
	6–9	12 (28.6)	12 (31.6)		7 (18.9)	3 (7.3)		1 (11.1)	2 (14.3)	
	10–19	12 (28.6)	9 (23.7)		13 (35.1)	18 (43.9)		4 (44.4)	3 (21.4)	
	≥20	5 (11.9)	2 (5.3)		5 (13.5)	3 (7.3)		1 (11.1)	4 (28.6)	

**Table 4:** Professional characteristics of the on-line survey respondents (students).

		Students		
		Trainee group *N* = 22	Control group *N* = 35	*Z* or Fisher; *p* values
Academic profile, *n* (%)	Junior college student	1 (4.6)	4 (11.4)	2.542; 0.493
	Undergraduate student	7 (31.8)	13 (37.1)	
	Graduate student (Master)	12 (54.6)	12 (34.3)	
	Graduate student (PhD)	2 (9.1)	6 (17.1)	

**Figure 2:**
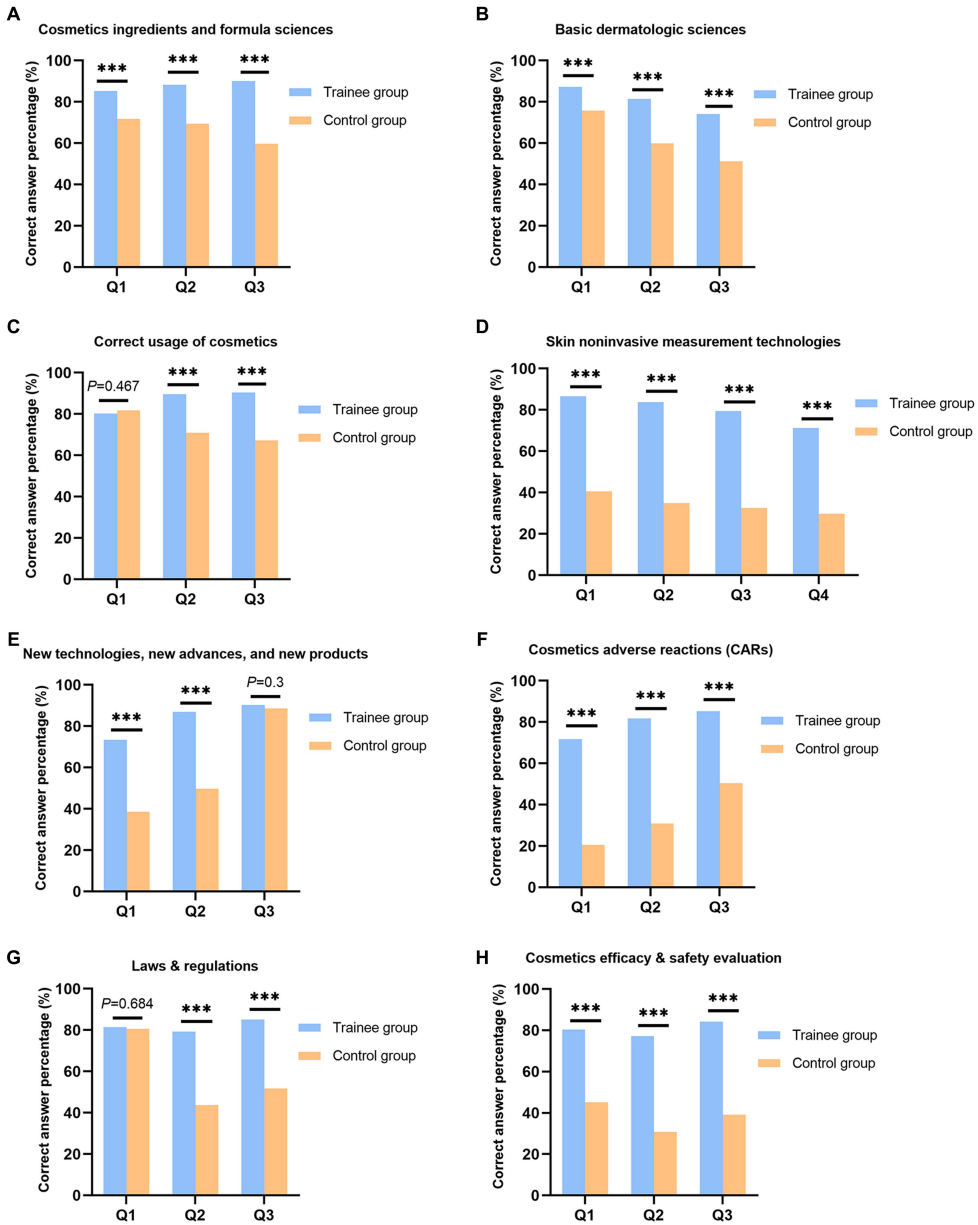
The correct answer percentage of the comprehensive test on cosmetic and dermatologic sciences, the trainee group vs. the control group. **(A)** cosmetics ingredients and formula sciences; **(B)** basic dermatologic sciences, dermatography, diagnosis and treatment of common skin diseases; **(C)** correct usage of cosmetics; **(D)** skin noninvasive measurement technologies; **(E)** new technologies, new advances, and new products; **(F)** cosmetics adverse reactions (CARs): identification, diagnosis, and treatment; **(G)** laws & regulations; and **(H)** cosmetics efficacy & safety evaluation, instruments introduction, study design, and subjects management (***: *p* = 0.000).

The comprehensive test on cosmetic and dermatologic sciences in the second part demonstrated that compared with the control group, the CME project significantly enhanced the trainees’ perception and knowledge regarding the cosmetics formula sciences, basic dermatologic sciences, cosmetics usage, noninvasive measurements, new advances, CARs, and laws (Figure 2). Notably, both the trainee group and the control group achieved similar correct answer percentage on 3 questions, regarding the functions of facial mask, new cosmetics preparation methods, and forbidden phrases for cosmetics packing and advertisements.

**Figure 3:**
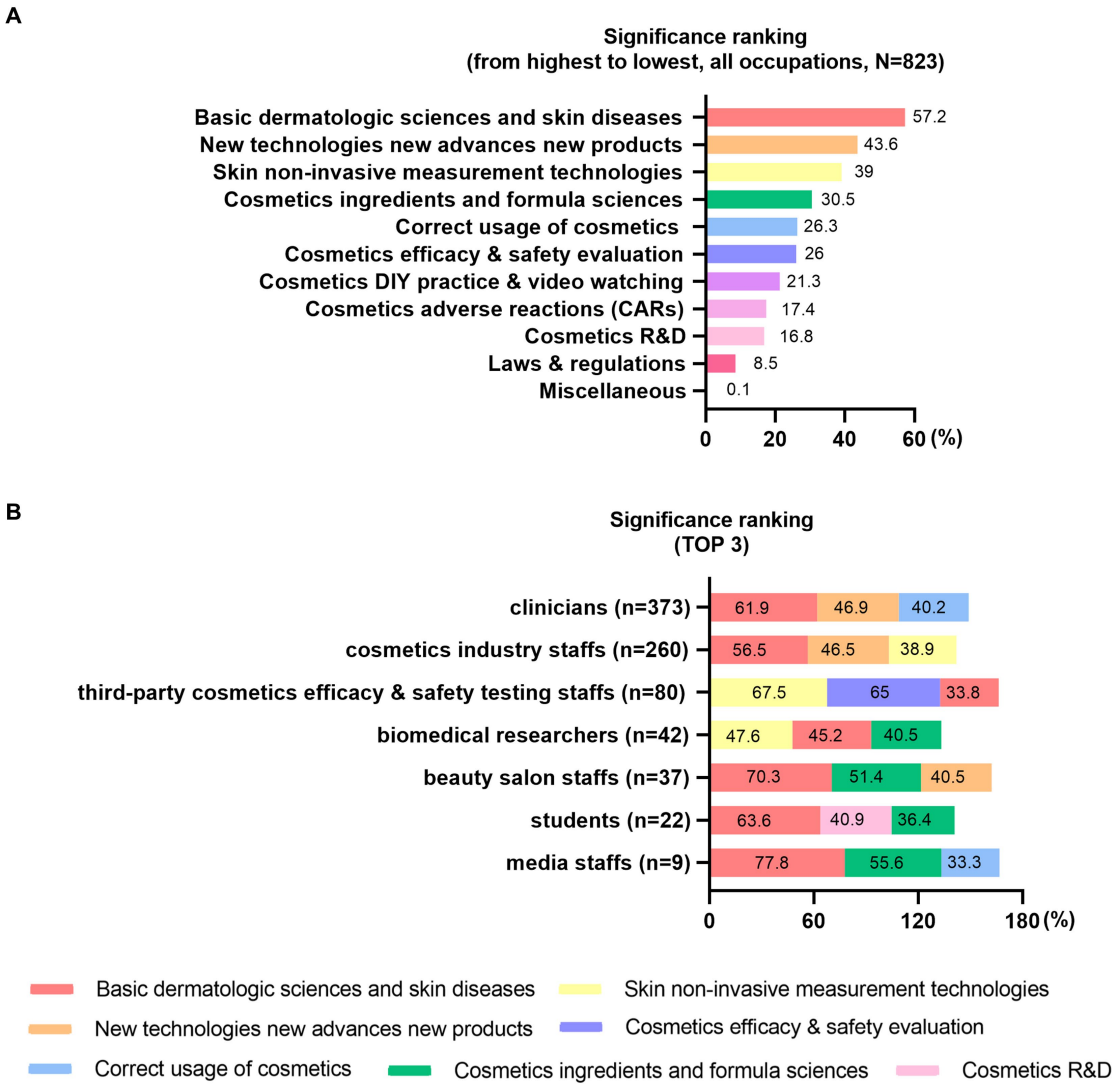
The significance ranking for the courses sections. **(A)** the significance ranking by trainees of all occupations and **(B)** the top 3 significance ranking by different occupations.

The third part of the survey revealed the detailed impact of the CME project. Most trainees (*n* = 707, 86%) from the past 5 years (2018–2022) completed the online survey. A considerable proportion of trainees learned this CME project on cosmetics sciences from “recommendation from the other people” (*n* = 302, 36.7%) and “the people who attended the CME before” (*n* = 201, 24.42%). Trainees of all occupations ranked “basic dermatologic sciences and skin diseases” as the most significant sections (Figure 3A). We noticed occupational variances in the top 3 significance ranking (Figure 3B).

**Figure 4:**
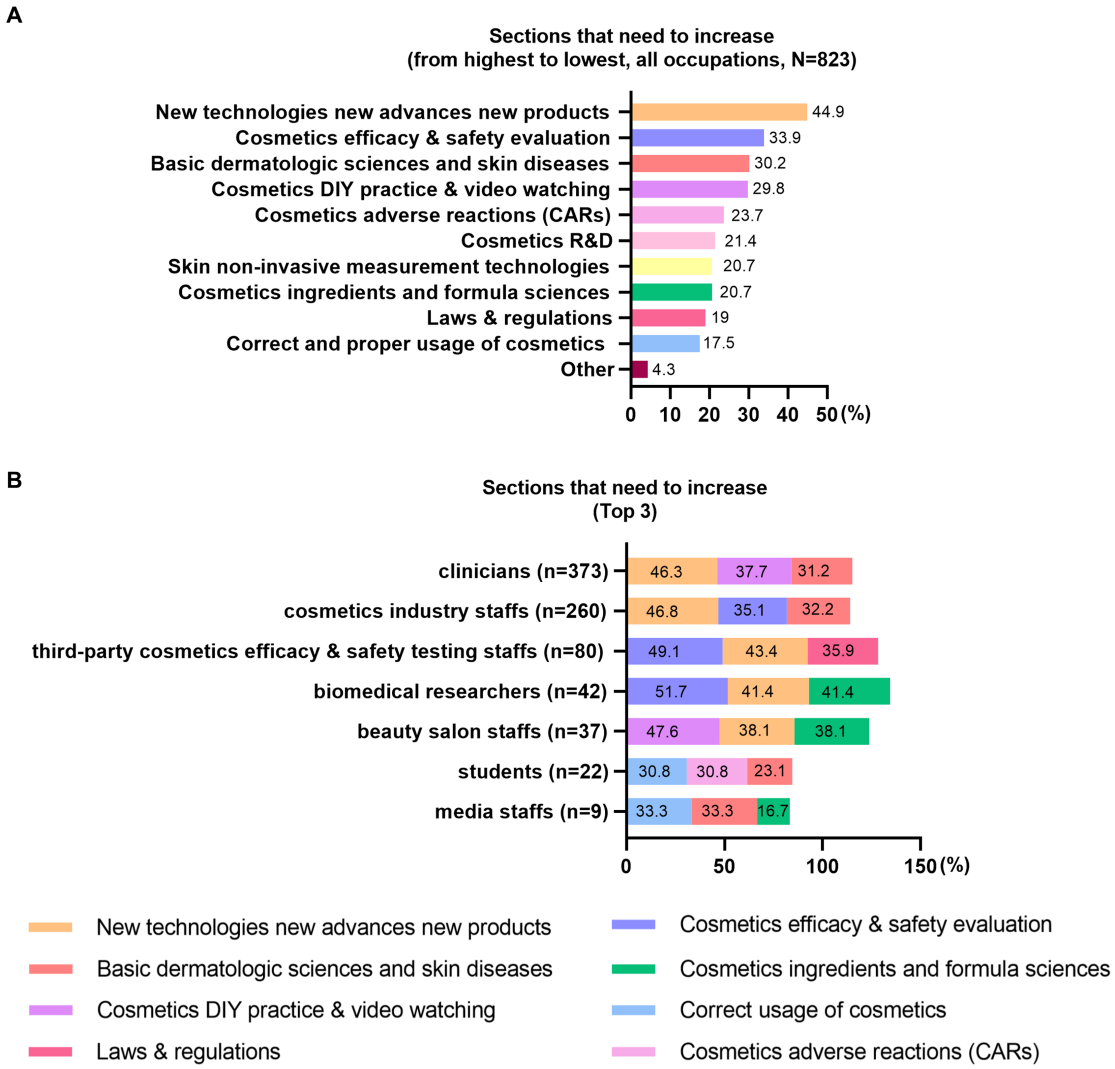
Sections that need to increase. **(A)** sections that need to increase, all occupations and **(B)** the top 3 sections that need to increase by different occupations.

**Figure 5:**
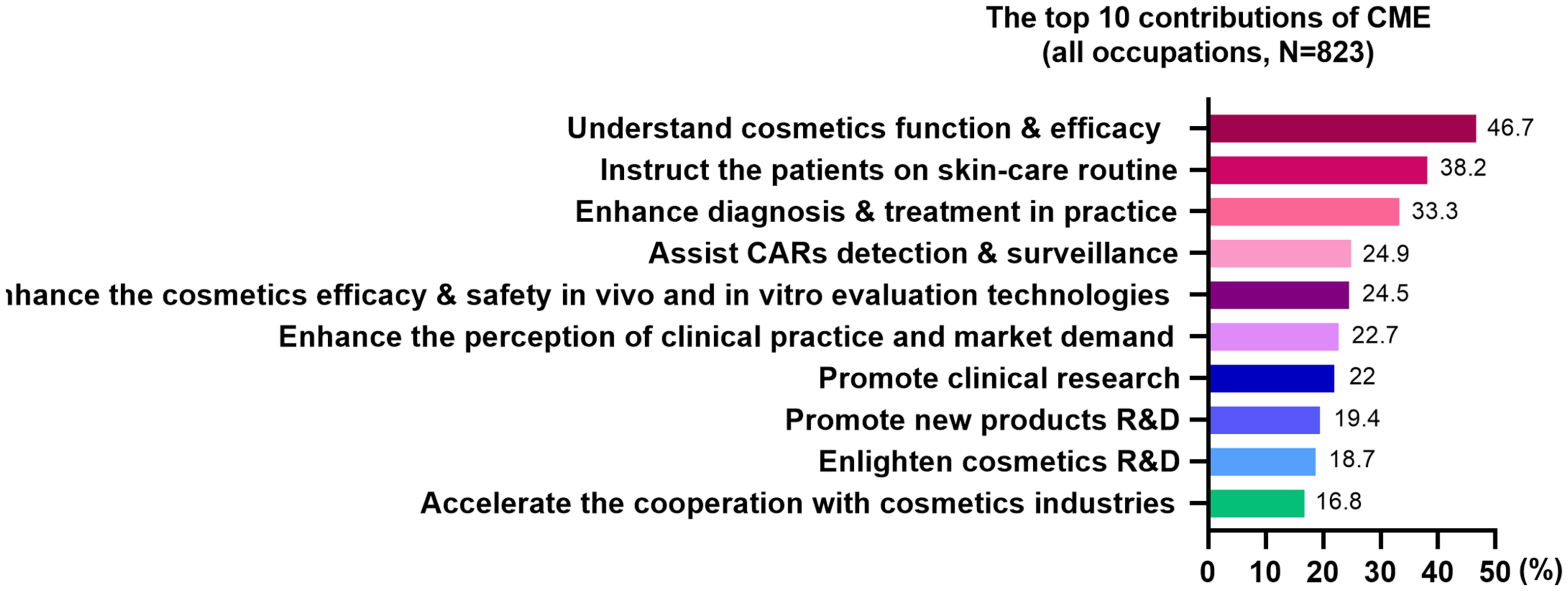
The top 10 contributions of the CME project, all occupations.

The majority of trainees (*n* = 537, 65.3%) wished to increase the course content (Figure 4A). The occupational variances were presented as well (Figure 4B). Additionally, some trainees expected to include the content on “clinical cases,” “the combination of medical aesthetic treatments and cosmetics,” “advances in dermatologic sciences,” and “pediatric cosmetics.” Trainees of all occupations believed the CME project has contributed most in “understand the function & efficacy of cosmetics” (Figure 5). The top 3 contributions varied based upon the occupations accordingly (Supplementary material 4). A few trainees stated that “the CME project instructed the industries and medical aesthetics private clinics on promotion and advertisements.” Over 97% (*N* = 823) of trainees were willing to recommend this CME project to the others.

## Discussion

The progress and prosperity in cosmetics have generated a gap between the cosmetics and their users. The “users” include not only regular customers, but also clinicians, industry personnel, researchers, testing agencies, beauty salon workers, and mass media. While versatile products and eye-catching advertisements offer seemingly endless options, not all users possess the ability to distinguish truth from falsehood. The gap remained as one major reason for inappropriate usage, insufficient efficacy, and even CARs. Education and training were necessary to bridge this gap as evidenced by the increasing number of participants in CME projects.

Compared with the majority of science popularization education projects, this CME had a noticeably smaller participant count. The objective of the CME project was not to educate the general population or ordinary individuals, but rather professionals in the field. It is now acknowledged that health promotion at the individual level has little effectiveness and tends to amplify socio-economic differences in health. The training and education for public health related professionals, however, might induce an amplification effect (29, 30). The professionals who undergo training through this CME project would subsequently educate and influence a large audience throughout their careers. Furthermore, the widespread geographic distribution of trainees suggested a potential national dissemination effect.

Despite clinicians potentially having the highest level of education among all involved occupations, they have not received sufficient training in cosmetic sciences. Cosmetic dermatology, as an emerging interdisciplinary science and a key subspecialty of academic dermatology, has not been included in the traditional compulsory curriculum of most medical schools in China. A significant proportion of dermatologists lack awareness or possess incorrect understanding about cosmetics. In recent years it has been increasingly realized that resident education in this field is essential for training dermatologists who are well-equipped to address the entire spectrum of patient needs (31, 32).

Beauty salon staffs are distinct from other professionals as they have access to people’s skin without requiring medical training or a license. However, the educational qualifications of most beauty salon staffs may not be sufficient for providing accurate skin care advice and treatments. Previous studies revealed that facial treatments at beauty salon more than once a week and using beauty salon products were positively correlated with the development of rosacea in Chinese population (18). Consistent with these findings, our online survey revealed that “basic dermatologic sciences and skin diseases” was ranked as the most significant sections by the beauty salon staffs. “Cosmetics DIY practice and video watching” was identified as the section that needs to increase most. Promoting education in dermatology and cosmetic sciences could help reduce beauty salon-related dermatoses and CARs.

The industrial staffs from various functional departments have enhanced and expanded their understanding in dermatologic and cosmetic sciences, which can be applied in cosmetics R&D as well as scientific interactions with clients. Research inspiration, industry collaboration, and improved perception of laws appeared meaningful for the third-party cosmetics testing staffs and biomedical researchers.

Notably, both groups achieved a comparable percentage of correct answers in relation to the functions of facial masks, methods for cosmetics preparation, and regulations on cosmetics packaging and advertising. This could be attributed to the increasing standardization of cosmetics manufacturing, packaging, and promotion. In addition, the public have spontaneously started accurate scientific information seeking. However, a comprehensive understanding and knowledge of cosmetic sciences necessitates systematic learning.

Public skin health promotion required concerted efforts. One of the most predominant characteristics of this CME project was multidisciplinary, which was demonstrated by the curriculum, the lecturers, and the trainees with multiple professional backgrounds. On the basis of an integrated content framework, the detailed content has been adjusted to advance with the times. The CME project offered the trainees the opportunities to meet people with a variety of professional backgrounds, education levels, and working experience. In other words, the project has built up a platform for acquaintance, communication, interaction, and cooperation. We deemed this platform establishment as one of the mechanisms to promote public skin health, in that the limited cooperation between occupations has hampered the cosmetic and dermatologic sciences development and restricted public health maintenance and promotion. This platform has also advocated industry-university-research institution cooperation and healthy competition among the cosmetics industries.

The trainees’ comments and feedback assisted us to identify some issues that deserved further consideration. For instance, some trainees expected to include the content on “pediatric cosmetics.” This might suggest some existing children’s skin health issues and a potential focus in future cosmetics R&D. How to guarantee the quality of online CME required further exploration as well.

There are several limitations to the present study. First, the questionnaire utilized in the study was not validated. The lack of validation may impact the reliability of the data collected through this instrument. Second, most of the online survey participants were the trainees from the last 5 years, rather than those from the earlier years. The possible reasons might be career move, or obscure memory of the training. Third, we did not perform the occupational sub-group analysis for the second part of the online survey due to the sample size of both groups. Fourth, the twenty-five single-choice items in the second section of the online survey only covered limited dermatologic and cosmetic sciences. Moreover, the trainee number of certain occupation (for instance, media staffs) was very small, and might not be adequately representative of the occupation.

## Conclusion

The CME project significantly enhanced the trainees’ cosmetic and dermatologic sciences, which bridged the gap between cosmetics and public skin health. This multidisciplinary CME also contributed to establishing an interdisciplinary interaction and cooperation platform for the multiple occupations involved in the public skin health maintenance and promotion.

## Data availability statement

The raw data supporting the conclusions of this article will be made available by the authors, without undue reservation.

## Ethics statement

The studies involving humans were approved by the ethical committee of West China hospital, Sichuan University. The studies were conducted in accordance with the local legislation and institutional requirements. The participants provided their written informed consent to participate in this study.

## Author contributions

YL: Conceptualization, Funding acquisition, Methodology, Writing – original draft, Writing – review & editing. XS: Data curation, Project administration, Writing – review & editing. WH: Methodology, Project administration, Writing – review & editing. XW: Project administration, Writing – review & editing.

## Footnotes

^1^ https://www.wjx.cn/
