# Supplementary material for: Promoting public skin health through a national continuing medical education project on cosmetic and dermatologic sciences: a 15-year experience
Source: Front Public Health. 2023 Nov 16;11:1273950. doi: 10.3389/fpubh.2023.1273950 (PMC10687160; doi:10.3389/fpubh.2023.1273950)
Supplement: Supplementary file 1 [file Data_Sheet_1.PDF]

PRELIMINARY SURVEY

Continuing Medical Education (CME)

On

Cosmetic and Dermatologic Sciences

Are you willing to attend an online survey on Cosmetic and Dermatologic Sciences CME project? You may need 40-45 minutes to complete the survey.

☐No.

☐Yes.

If yes, please answer the following questions:

Occupation:

A Clinicians: ☐Public hospital ☐Private practice

Specialization: ___________________

B Cosmetics industry staff: ☐R&D staff ☐Efficacy & safety evaluation staff

☐Sales staff ☐Management staff

C Biomedical researchers

D Third-party cosmetics efficacy & safety testing staff

E Beauty salon staff

F Students: ☐Junior college student ☐Undergraduate student

☐Graduate student (Master) ☐Graduate student (PhD)

Specialization: ___________________

G Media staff: ☐Journals and periodicals ☐Media anchor

☐Other: ___________________

H Other (please specify): ___________________

Education level

☐Below college diploma ☐College diploma ☐Bachelor's degree

☐Master's degree ☐Doctor's degree

Work experience (yrs):

☐$\leqslant 5$ ☐6-9 ☐10-19 ☐$\geqslant 20$

Professional title:

☐N/A ☐Primary ☐Middle ☐Senior
