# Supplementary material for: Promoting public skin health through a national continuing medical education project on cosmetic and dermatologic sciences: a 15-year experience
Source: Front Public Health. 2023 Nov 16;11:1273950. doi: 10.3389/fpubh.2023.1273950 (PMC10687160; doi:10.3389/fpubh.2023.1273950)
Supplement: Supplementary file 2 [file Data_Sheet_2.PDF]

ONLINE SURVEY

Continuing Medical Education (CME)

On

Cosmetic and Dermatologic Sciences

This survey contains three parts. Part one and Part two are for both the trainee group and the control group. Part three is for the trainee group ONLY.

PART ONE

Demographic and Professional Information

For both the trainee group and the control group

Occupation:

A Clinicians: ☐Public hospital ☐Private practice

Specialization: ___________________

B Cosmetics industry staff: ☐R&D staff ☐Efficacy & safety evaluation staff

☐Sales staff ☐Management staff

C Biomedical researchers

D Third-party cosmetics efficacy & safety testing staff

E Beauty salon staff

F Students: ☐Junior college student ☐Undergraduate student

☐Graduate student (Master) ☐Graduate student (PhD)

Specialization: ___________________

G Media staff: ☐Journals and periodicals ☐Media anchor

☐Other: ___________________

H Other (please specify): ___________________

Gender:

☐Male ☐Female

Education level

☐Below college diploma ☐College diploma ☐Bachelor’s degree

☐Master’s degree ☐Doctor’s degree

Age (y/o):

☐≤30 ☐31-39 ☐41-49 ☐≥50

Work experience (yrs):

☐≤5　☐6-9　☐10-19　☐≥20

Professional title:

☐N/A　☐Primary　☐Middle　☐Senior

PART TWO

Comprehensive Test on Cosmetic and Dermatologic Sciences (Single Choice)

For both the trainee group and the control group

*** Please complete this part within 25 minutes.**

Cosmetics ingredients and formula sciences

1. The ingredients of sunscreen products are ______

   A Titanium dioxide

   B Zinc oxide

   C Octyl methoxycinnamate

   D All above

2. Which of the following is a forbidden anti-comedone cosmetic ingredient?

   A Salicylic acid

   B Antibiotics

   C Extracts of traditional Chinese medicine

   D Retinol

3. Which of the following is not preservative in cosmetics?

   A Benzyl alcohol & benzaldehyde

   B Benzoic acid

   C Salicylic acid

   D Arbutin

Basic dermatologic sciences, dermatography, diagnosis and treatment of common skin diseases

1. The pathogenesis of melasma includes______

   A Skin barrier damage

   B Inflammatory cytokines

   C Capillary proliferation and angiotelectasis

   D All above

2. Which of the following is associated with skin barrier damage?

   A Sensitive skin

   B Acne & rosacea

   C Atopic dermatitis

   D All above

3. Which Fitzpatrick types are susceptible to hyperpigmentation induced by bule light irradiation?

   A Type I and type II

   B Type II and type III

   C Type III and type VI

   D Type VI and type V

Correct usage of cosmetics

1. The functions of facial mask include ______

   A Promoting facial blood circulation

   B Absorption, exfoliation, cleaning

   C Moisturizing

   D All above

2. PA+++ means ______

   A PA value 2-3

   B PA value 3-4

   C PA value 4-5

   D PA value ≥8

3. Which type of cosmetics is not recommended for cases with sensitive skin?

   A Sun screen cosmetics

   B Exfoliating cosmetics

   C Moisturizers

   D Soothing cosmetics

Skin noninvasive measurement technologies

1. The non-invasive measurements for dermo-microcirculation include ______

   A Laser Doppler blood flow instrument

   B Transdermal oxygen partial pressure detector

   C Capillary scope

   D All above

2. TEWL represents ______

   A One key parameter of skin barrier function

   B The moisture content of stratum corneum

   C The sebum production

   D The skin roughness

3. The influencing factors of skin non-invasive measurements include ______

   ①the ambient environment ②the subject's position

   ③the diet before the measurements ④whether it is the same operator

   A ①②③④ B ①④ C ①②④ D ②③④

4. Which one is wrong about TEWL measurements?

   A The higher the TEWL is, the better the stratum corneum barrier function would be

   B An obvious TEWL decrease after the moisturizer application implies significant moisturizing effects

   C Both enclosed type and open type probes are available

   D The subjects are supposed to rest for 15-30 minutes before the measurements

New technologies, new advances, and new products

1. Which one is wrong about hair transplantation?

   A Frequently-used hair transplantation methods include FUT and FUE

   B FUT may result in linear scars

   C FUE does not result in scars

   D Generally medication treatment is needed following hair transplantation

2. The advantages of lyophilized facial mask include ______

   A Freeze-dried preservation contributes to a long-lasting effect

   B Reduces the content of preservatives

   C Being portable and convenient

   D All above

3. Which of the following is a new cosmetics preparation method?

   ①liposome ②microemulsion ③microneedle ④nanocarriers

   A ①②③

   B ②③④

   C ①②④

   D ①②③④

Cosmetics adverse reactions (CARs): identification, diagnosis, and treatment

1. Cosmetic group adverse reactions represent the adverse events caused by the same type of cosmetics manufactured by one enterprise, which involve ______ and require emergency management.

   A Adverse reactions ⩾5 cases, serious adverse reactions ⩾2 cases

   B Adverse reactions ⩾5 cases, serious adverse reactions ⩾3 cases

   C Adverse reactions ⩾10 cases, serious adverse reactions ⩾5 cases

   D Adverse reactions ⩾10 cases, serious adverse reactions ⩾3 cases

2. The practice standard of an open patch test is ______

   A Test substances application once per day for continuous 7 days

   B Test substances application twice per day for continuous 7 days

   C Test substances application once per day for continuous 14 days

   D Test substances application twice per day for continuous 14 days

3. The principle of cosmetic dermatosis diagnosis includes ______

   ①cosmetics contact history ②skin lesions developed in the cosmetics application area ③exclude non-cosmetic factors ④patch test results

   A ①②③ B ②③④ C ①③④ D ①②③④

Laws & regulations

1. The forbidden phrases for cosmetics external packing and advertisements include ______

   A Therapy

   B Anti-bacteria

   C Detoxification

   D All above

2. Which of the following are exempt from efficacy evaluation?

   ①cleaning products ②makeup removers

   ③fragrance products ④anti-wrinkle products

   A ①②③ B ②③④ C ①③④ D ①②③④

3. If the SPF value decreases over ______ of the claimed or anticipated one, this product will not be labelled as “water-proof”.

   A 30%

   B 40%

   C 50%

   D 60%

Cosmetics efficacy & safety evaluation, instruments introduction, study design, and subjects management

1. The efficacy evaluation of hair growth includes ______

   ①hair density ②hair diameter ③hair growth rate ④anagen/telogen ratio

   A ①②③ B ②③④ C ①③④ D ①②③④

2. For the labelled waterproof sunscreen products, the SPF value should be the one after ______ minutes’ water resistance test.

   A 20

   B 30

   C 40

D 50

3. The efficacy evaluation of freckle-removing cosmetics does not include ______

   A Tyrosinase activity measurement

   B WOOD light examination

   C Melanin content measurement

   D Trichromatic analysis

PART THREE

Your Comments on the CME project

For the trainee group ONLY

1. **When did you attend the CME project?**

A 2008	B 2009	C 2010	D 2011	E 2012
F 2013	G 2014	H 2015	I 2016	J 2017
K 2018	L 2019	M 2020	N 2021	O 2022

2. **How did you learn the CME project?**

A Journals and periodicals

B The CME project official account

C Academic conference

D Recommendation from the other people

E The people who attended the CME before

F Other: ___________________

3. **Which section(s) do you consider most significant? (up to 3 items)**

A Cosmetics ingredients and formula sciences

B Basic dermatologic sciences, dermatologic imageology, diagnosis and treatment of common skin diseases

C Correct and proper usage of cosmetics

D Skin noninvasive measurement technologies

E Cosmetics DIY practice & video watching

F New technologies, new advances, and new products

G Cosmetics research & development (R&D)

H Cosmetics adverse reactions (CARs): identification, diagnosis, and treatment

I Laws & regulations

J Cosmetics efficacy & safety evaluation, instruments introduction, study design,

and subjects management

K Other (please specify): _________________

4. **Does the content of this CME project need to increase?**

A No

B Yes, please specify (up to 3 items)

☐Cosmetics ingredients and formula sciences

☐ Basic dermatologic sciences, dermatologic imageology, diagnosis and treatment of common skin diseases

☐Correct and proper usage of cosmetics

☐Skin noninvasive measurement technologies

☐Cosmetics DIY practice & video watching

☐New technologies, new advances, and new products

☐Cosmetics research & development

☐CARs: identification, diagnosis, and treatment

☐Laws & regulations

☐Cosmetics efficacy & safety evaluation, instruments introduction, study design, and subjects management

☐Other (please specify): _________________

5. **How does the CME project contribute to your work or study? (multiple choice, occupation categories for reference only)**

Clinician

☐Understand cosmetics function & efficacy

☐Instruct the patients on skin-care routine

☐Inspire clinical research

☐Enhance diagnosis & treatment in practice

☐Promote new products R&D

☐Accelerate the cooperation with cosmetics industries

☐Assist CARs detection & surveillance

Cosmetics industry R&D staff

☐Enhance the perception of clinical practice and market demand

☐Inspire novel materials research

☐Inspire formula design

☐Enlighten cosmetics R&D

☐ Enhance cosmetics efficacy & safety *in vivo* and *in vitro* evaluation technologies

Cosmetics industry sales staff

☐Improve the acquaintance of cosmetics ingredients

☐Promote sales skills

☐Assist sales staff training

☐Facilitate the communication with clinicians

☐Facilitate the communication with customers

☐Assist CARs identification & management

Cosmetics industry management staff

☐Enhance the awareness of the laws & regulations on cosmetics trademark & advertisements

☐Assist the understanding of cosmetics efficacy & safety evaluation reports

☐Standardize cosmetics efficacy & safety evaluation

☐Facilitate the cooperation with clinicians on cosmetics R&D

Biomedical researchers

☐Inspire scientific research

☐Enhance dermatologic and cosmetic sciences teaching level

☐Improve personal skin-care routine

☐ Inspire the cooperation with industries on research achievements transformation

Third-party cosmetics efficacy & safety testing organization staff

☐Improve subjects management

☐Inspire cosmetics efficacy & safety evaluation design

☐Understand instruments rationales and standardize instruments operation

☐Enhance the perception of laws & regulations

☐Standardize the writing of cosmetics efficacy & safety evaluation reports

Beauty salon staff

☐Increase basic dermatologic sciences

☐Increase scientific skin-care knowledge

☐Facilitate the communication with customers

☐Standardize the skin-care operation

☐Inspire business startups

Students

☐Inspire future career planning

☐Inspire clinical & basic sciences research design

☐Inspire future specialty selection

Other (please specify): _________________

6. **Will you recommend this CME project to the others?**

A Absolutely not

B Probably not

C Not sure

D Probably yes

E Absolutely yes
