# Supplementary material for: Promoting public skin health through a national continuing medical education project on cosmetic and dermatologic sciences: a 15-year experience
Source: Front Public Health. 2023 Nov 16;11:1273950. doi: 10.3389/fpubh.2023.1273950 (PMC10687160; doi:10.3389/fpubh.2023.1273950)
Supplement: Supplementary file 3 [file Data_Sheet_3.PDF]

The age and gender distribution of the trainee group and control group

		Trainee group (N=823)	Control group (N=586)	*P* values
Gender, n (%)	Male	210 (25.5)	123 (21)	0.049
	Female	613 (74.5)	463 (79)	
Age (y/o), n (%)	≤30	207 (25.2)	124 (21.2)	0.096
	30-39	366 (44.5)	249 (42.5)	
	40-49	194 (23.6)	166 (28.3)	
	≥50	56 (6.8)	47 (8)	
