# Supplementary figures and images for: Promoting public skin health through a national continuing medical education project on cosmetic and dermatologic sciences: a 15-year experience
Source: Front Public Health. 2023 Nov 16;11:1273950. doi: 10.3389/fpubh.2023.1273950 (PMC10687160; doi:10.3389/fpubh.2023.1273950)

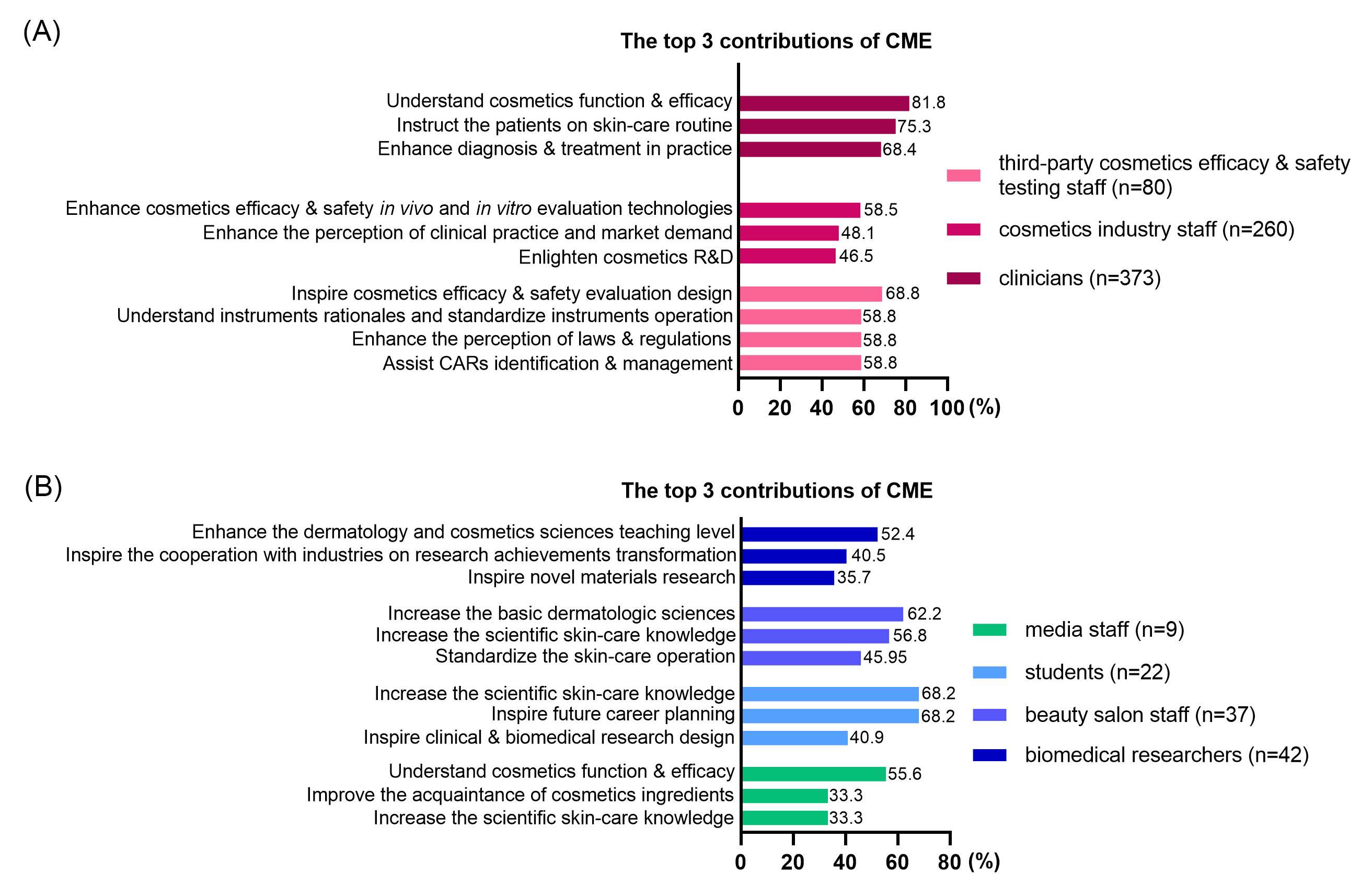

Supplement: Supplementary file 4 [file Image_1.TIF]
